# The Interrelationship of Adenomatoid Odontogenic Tumour and Dentigerous Cyst: A Report of a Rare Case and Review of the Literature

**DOI:** 10.1155/2012/358609

**Published:** 2012-12-12

**Authors:** Anshita Agarwal, K. Y. Giri, Sarwar Alam

**Affiliations:** ^1^Department of Oral Pathology, Vananchal Dental College and Hospital, Garhwa, Jharkhand 822114, India; ^2^Department of Oral & Maxillofacial Surgery, Institute of Dental Sciences, Bareilly 243006, India

## Abstract

The adenomatoid odontogenic tumor (AOT) is a relatively uncommon lesion which mainly affects females in their second decade of life, exhibiting predilection for the anterior region of the maxilla. The lesion is usually associated with the crown of an enclosed tooth, most commonly the maxillary canine. In this paper we present a case of adenomatoid odontogenic tumor associated with a dentigerous cyst affecting the left maxilla in a 15-year-old female. We also discuss clinical, radiographic, histopathologic, and therapeutic features of the case.

## 1. Introduction 

Adenomatoid odontogenic tumor (AOT) is comprised of odontogenic epithelium in a variety of histoarchitectural patterns, embedded in a mature connective tissue stroma and characterized by slow but progressive growth. In the WHO classification of 2005, AOT is included under “odontogenic epithelium with mature, fibrous stroma without odontogenic ectomesenchyme” [1]. The age range in which AOT occurs varies between 3 and 82 years. More than two-thirds are diagnosed in the second decade of life and 90% are found before the age of 30. More than half of the cases occur among teenagers. The male : female ratio is 1 : 1.9 [2, 3]. In some Asian countries the ratio may reach 1 : 3.2. It almost exclusively occurs intraosseously with a preference for the maxilla over the mandible with a ratio of 2.1 : 1 [4]. The rare peripheral type occurs almost exclusively in the anterior maxillary gingiva. Intraosseous AOT may be found in association with unerupted permanentteeth (follicular type), in particular the four canines that account for 60% with the maxillary canines alone accounting for 40%.

 We hereby present the first case of AOT that originated in the wall of a dentigerous cyst of the maxilla in pediatric age group in the Indian subcontinent.

## 2. Case Report

**Figure 1:**
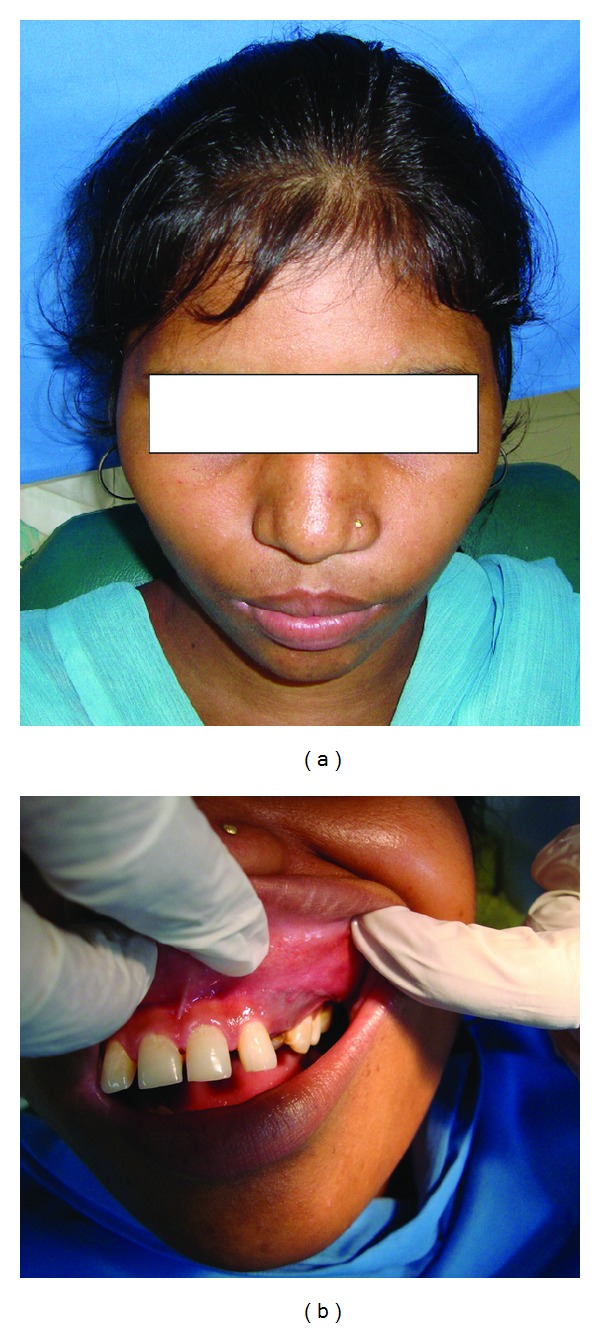
(a) Preoperative extraoral photograph of 15-year-old female with swelling on left maxilla. (b) Preoperative intraoral photograph showing missing canine tooth.

**Figure 2:**
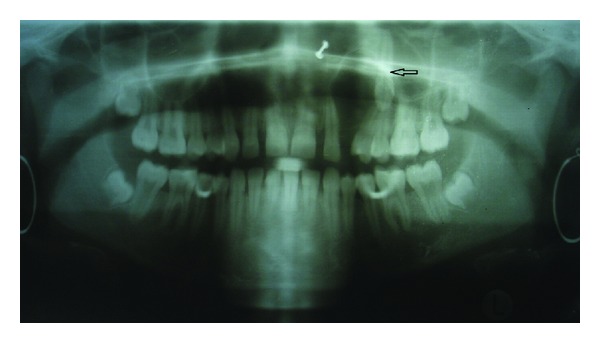
Oral orthopantomogram (OPG) showing well-defined radiolucent cyst (arrow) with canine tooth.

A 15-year-old Indian female was referred by her general practitioner for evaluation of a maxillary swelling to Department of Oral and Maxillofacial Surgery, Career Post Graduate Institute of Dental Sciences & Hospital, Lucknow. The medical history was insignificant. The patient was asymptomatic and in good general health. Intraoral examination disclosed a nontender expansion of the left maxilla with missing canine tooth. Covering mucosa appeared normal (Figures 1(a) and 1(b)). The patient had no nerve deficit or adenopathy in the face or neck region. An orthopantomogram revealed a well-defined, unilocular radiolucency in maxilla with expansion and thinning of all its bony walls with the left upper canine tooth (Figure 2). It also showed displacement of tooth and root resorption of first and second premolars. A Denta scan (64 slice CT Scan) showed a well-defined tumor mass covering the complete left maxilla. According to the clinical and surgical findings, the lesion was diagnosed as a dentigerous cyst. Enucleation of the lesion was performed under local anesthesia, to completely extirpate the cystic lesion with involved impacted upper left canine. 

**Figure 3:**
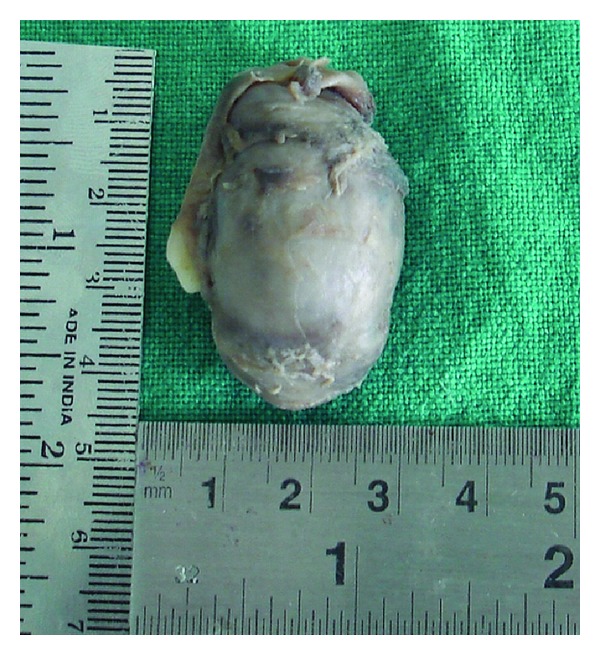
Surgical gross revealing a cystic lesion with embedded canine.

**Figure 4:**
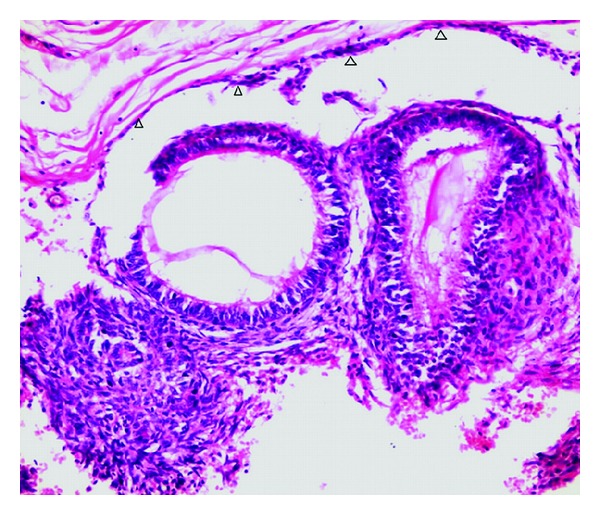
Duct-like structures of odontogenic epithelium; one filled with eosinophilic material, along with 1-2 cell layer think of cuboidal cells (arrows) (H&E ×40).

**Figure 5:**
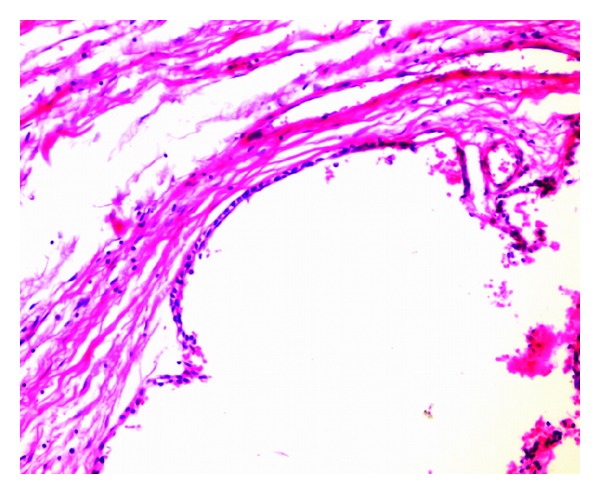
Cystic area lined by 1-2 cell layers thick of cuboidal cells (H&E, ×10).

The differential diagnosis of dentigerous cyst, calcifying odontogenic cyst, calcifying epithelial tumor, keratocystic odontogenic tumor, and unicystic ameloblastoma was made. The surgical gross measured 4.5 cm × 2.5 cm × 4 cm with a smooth surface and was associated with the canine (Figure 3). No calcifications were present in the cystic lumen. Microscopically, sections revealed variable sized solid nodules of columnar cells and spindle-shaped cells of odontogenic epithelium forming nests and rosette-like structures. Eosinophilic amorphous material was present between the duct-like spaces which were lined by a single row of columnar epithelial cells, with the nuclei polarized away from luminal surface (Figure 4). The cystic area was composed of dense fibrous tissue lined by one to three layers of non keratinized stratified squamous epithelium (Figure 5). Furthermore, this lining of the cyst was in continuity with the AOT area. Consequently, the final histological diagnosis of AOT arising from a dentigerous cyst over the left maxilla was made. The postoperative course was uneventful and for the past 3 years there has been no sign of recurrence at follow-up.

## 3. Discussion

AOT was first described by Steensland in 1905 [12]. However, a variety of terms have been used to describe this tumor. Unal et al. [13] produced a list containing all nomenclatures for AOT reported in the literature. Many different names like adenoameloblastoma, ameloblastic adenomatoid tumor, adamantinoma, epithelioma adamantium, or teratomatous odontoma have been used before to define this lesion. In 1999, Philipsen and Reichart [14] presented a review based on reports published until 1997 which showed some interesting aspects regarding epidemiological figures of this tumor [15]. Most recently, Esquiche Leon et al.described a multicentre study of both the clinicopathological and immunohistochemical features of 39 cases of AOT [16].

Rick has reported AOT to occur with many types of cysts and neoplasms including dentigerous cyst, calcifying odontogenic cyst, odontoma, and ameloblastoma. In relation with a dentigerous cyst the AOT may demonstrate, grossly and microscopically, one or more associated cystic cavities. Some of these cysts are lined by nonkeratinized stratified squamous epithelium which is similar to the lining of the dentigerous cyst or lined by less structured membrane that may demonstrate bud-like extensions into the connective tissue [17]. In our case, cystic area was composed of dense fibrous tissue lined by one to three layers of nonkeratinized stratified squamous epithelium.

In our case, AOT and dentigerous cyst are found in the same lesion. Clinical, radiographic, and macroscopic findings in the present case are consistent with descriptions of the lesion in the dental literature [17]. AOT is usually solid but is occasionally cystic. Very few cases have been described that arise in association with a dentigerous cyst. A systematic search of the English language medical literature revealed only nine such cases including our own in the pediatric population, and only one case from India (Table 1).

The structure of the cyst and its insertion around the crown of an unerupted tooth were typical of a dentigerous cyst. Odontogenesis is a complex process and neoplastic or hamartomatous lesions can occur at any stage of odontogenesis. The secondary development of an ameloblastic proliferation, whether hyperplastic or neoplastic, is well known but remains controversial. In this case, the multifocal cellular proliferation had the structure of an AOT. Its mural development in a dentigerous cyst is not uncommon. The tumor is well encapsulated and shows an identical benign behavior. Therefore, conservative surgical enucleation produces excellent outcome without recurrence. 

Very few case reports of AOT arising from a dentigerous cyst with histological identification have previously been reported. The present case leaves us with an ambiguity whether we should consider it as an odontogenic cyst, containing both epithelial and mesenchymal components or a hybrid tumor.

## Figures and Tables

**Table 1:** A systematic Clinical data of the reported cases of adenomatoid odontogenic tumor arising from a dentigerous cyst in pediatric age group till date.

Authors	Age/sex	Race	Site	X-ray finding	Other finding
(1) Valderrama [5]	16/female	Philippians	Maxilla	Unilocular radiolucency, surrounding tooth 14 crown	Presence of complex odontoma
(2) Warter et al. [6]	8/male	Nigerian	Maxilla	Unilocular radiolucency, surrounding tooth 13 crown	Contained melanocytes and melanin-laden epithelial cells
(3) Tajima et al. [7]	15/male	Japanese	Maxillary antrum	A well-defined radiopaque mass and crown of unerupted 28	—
(4) Garcia-Pola Vallejo et al. [8]	12/male	Spanish	Maxilla	Unilocular radiolucency, surrounding tooth 23 crown	Agenesis of tooth 15 and 24
(5) Bravo et al. [9]	14/male	Notstated	Maxilla	Unilocular radiolucency, surrounding tooth 23 crown	Expanding to sinus
(6) Nonaka et al. [10]	13/female	Brazil	Maxilla	Unilocular radiolucency with few radiopaque areas 23 and 24	—
(7) Chen et al. [11]	15/male	Tiwan	Maxilla	Radiolucency around upper deciduous canine	Odontoma-like areas were observed
(8) Khot and Vibhakar [18]	17/female	Indian	Mandible	Unilocular radiolucency surrounding unerupted 33	Involving whole ramus with pus discharge
(9) Our case	15/female	Indian	Maxilla	Unilocular radiolucency, tooth 23 crown surrounded	Root resorption of adjacent teeth
